# Knowledge toward quality improvement among Jordanian nursing students: A cross-sectional study

**DOI:** 10.1371/journal.pone.0311786

**Published:** 2024-10-08

**Authors:** Saleh Al Omar, Anas H. Khalifeh, Bahaaeddin M. Hammad, Zakaria M. Abdelrahim

**Affiliations:** 1 Faculty of Nursing, Al-Balqa Applied University, Salt, Jordan; 2 Department of Community & Mental Health Nursing, Faculty of Nursing, Zarqa University, Zarqa, Jordan; 3 Faculty of Nursing, Arab American University, Jenin, Palestine; 4 Infection Control Office, Jordan University Hospital, Amman, Jordan; The World Islamic Sciences and Education University, JORDAN

## Abstract

**Background:**

Nurses have a critical role in quality improvement (QI) and patient safety. This necessitates gaining knowledge and mastering QI abilities to lead and participate in QI programs in clinical practice.

**Aim:**

To assess undergraduate nursing students’ knowledge about QI, and experience of patient safety situation; and examine the relationship between obtaining information on healthcare quality and knowledge about QI in Jordan.

**Design:**

A descriptive cross-sectional correlational design was used. The study engaged undergraduate nursing students from two Jordanian universities (Public and private universities). A convenient sample of 147 nursing undergraduate students from universities.

**Methods:**

Data were collected using paper-based self-reported questionnaires. The Quality Improvement Knowledge, Skills, and Attitudes (QulSKA) survey and the Healthcare Professional Patient Safety Assessment Curriculum Survey (HPPSACS) were used to assess knowledge about QI and patient safety.

**Results:**

Out of 152 students, 147 completed the study. Overall, QI knowledge was moderate (mean score 57.7). Students from the private university scored significantly higher than public university students. Regarding patient safety, 74.8% of students observed medical errors in clinical areas. However, only 57.1% of the students disclosed a medical error to a faculty member. Also, the results showed a significant positive correlation between knowledge level about QI and obtaining information about QI, quality in healthcare, and patient safety from sources other than the undergraduate nursing program at universities (*p*≤.001).

**Conclusions:**

This study suggests a potential gap in QI education. Integrating QI concepts into nursing curricula may be necessary to prepare future nurses for healthcare quality improvement.

## Introduction

Since the 2000s, attention has been focused more on improving the quality of healthcare systems, prompting the development of new paradigms for quality improvement (QI) [1]. Quality improvement (QI) and patient safety are concerning topics for healthcare systems worldwide and in Jordan [2–5]. Patient safety employs organized activities to reduce risks, errors, and avoidable harm that may occur in healthcare systems [6]. However, despite improving healthcare’s effectiveness, it is becoming more complicated due to the increased use of new technologies, medications, and treatments [7].

Medical errors can threaten patient safety [8]. In the United States (US), medical errors remain the third leading cause of death [9]. Medical errors may also lead to increased healthcare expenses and readmission rates [10]. However, by using QI principles, medical errors can be minimized and their harmful effects avoided [10]. This could result in a $108 million reduction in healthcare expenses [11]. Delivering high-quality healthcare is critical. The fundamental ethical value of healthcare is to do no harm, as emphasized by Florence Nightingale and the World Health Organization [7]. Today’s healthcare systems rely significantly on healthcare workers to ensure patient safety. Despite significant changes in healthcare systems, they are obliged to provide safe and high-quality care [1].

Nurses make up the majority of the healthcare workforce and provide direct patient care around the clock. However, they may lack the necessary skills and knowledge to promote patient safety and are prone to making mistakes in complicated clinical situations, which may harm both patients and students [12]. Safe patient care provided by nurses can be enhanced by adequate preparation and education of nurses during their undergraduate education [13]. Nevertheless, QI is poorly integrated into nursing education and associated curricula [14].

There is a gap between theory and practice, which leads to inadequate application of nursing students’ knowledge regarding patient safety and quality of care [15]. Newly graduated nurses may face challenges in integrating ideas of patient safety and quality of care into practice. These challenges could be associated to a lack of competency in these concepts during undergraduate study and a lack of involvement of quality specialists in educating newly hired staff about quality and patient safety [14]. Since patient safety and QI have become increasingly important globally, nursing schools in Jordan have begun including patient safety courses in their curricula. However, teaching techniques differ greatly depending on the nursing school and the course length.

In Jordan, 71.4% of undergraduate nursing students reported they had not received any training in preventing and reporting medical errors (N = 303) [2]. Nevertheless, the study focused on medical errors only and did not consider other factors that may threaten patient safety. Another study indicated that undergraduate nursing students had moderate satisfaction with their competencies and knowledge concerning patient safety (N = 279) [16]. However, the researcher did not employ a representative sample of students from private universities, and the main focus was patient safety only, not QI. Nursing educators emphasize strengthening undergraduate students’ clinical practice skills; nevertheless, patient safety and quality in healthcare education get less attention. Most Jordanian universities do not have standalone patient safety and healthcare quality courses in the curricula of undergraduate nursing programs. Little is known regarding Jordanian undergraduate nursing students’ views on patient safety and quality in healthcare [16]. Therefore, this study aimed to assess undergraduate nursing students’ knowledge level about QI and patient safety practices in Jordan. Additional two secondary objectives were to examine the relationship between the level of knowledge about QI and the source of obtaining information about QI and healthcare quality in Jordan. In addition, to assess the difference in knowledge level about QI between nursing undergraduate students at two different universities.

## Methods

### Study design, sample, and settings

A descriptive cross-sectional correlational design was used to enroll a convenient sample of nursing students studying at two universities in Jordan that use the English language as an instruction mode. Eligibility criteria included being above 18 years, an undergraduate nursing student at the second, third, or fourth-year level, read and understand the English language, and agreeing to participate in the study. However, students from the bridging nursing program were excluded as they might have prior experience with quality in healthcare. The sample size was calculated using G*Power software [17]. A power analysis was performed using the correlation test with power at 0.95 with alpha set at 0.05 and medium effect size. The estimated sample size was 138 participants. To compensate for possible missing data or incomplete questionnaires and possible nonresponse rate, this number increased to 152 participants.

The study was conducted at two Jordanian universities with nursing schools in the middle of Jordan: public and private between July 7, 2022 and Aug 29, 2022. The researchers selected these universities because they are large universities located in the two cities where more than half of the country’s population lives and their nursing faculties have the highest number of nursing students.

### Instruments

The questionnaire was composed of three instruments and administered in the English version. The researchers developed the first instrument. It sought information about students’ demographic characteristics. The second instrument was the Quality Improvement Knowledge, Skills, and Attitudes (QulSKA), which was used to assess the students’ knowledge level about QI. The original QulSKA has 17, 45, and 11 items for measuring knowledge, skills, and attitudes, respectively. However, the researchers adopted the subscale of "knowledge", which included 17 multiple-choice items and had the highest possible score of 100 [18]. The number of correct answers on the knowledge subscale of the QulSKA was summed, and the percentage of correct items (out of a possible 100%) was calculated for each participant.The original authors described a knowledge level of 69.2 as a strong knowledge level [18]. It was used among nursing students [18, 19] and nursing educators [20]. The inter-item correlation coefficient of the QulSKA was 0.839 [18] and 0.93 [19]. In the present study, Cronbach’s alpha was 0.77.

The third instrument was the Healthcare Professional Patient Safety Assessment Curriculum Survey (HPPSACS). It is a 22‐item adapted by Chenot and Daniel [20] and tested among 318 undergraduate nursing students from seven nursing colleges. The HPPSACS instrument was designed to assess students perceived knowledge, skills, and attitudes towards patient safety. It consists of 18 items that measure attitudes about patient safety, five items that measure the comfort of performing skills related to patient safety using a five-item Likert scale ranging from 1 (strongly disagree) to 5 (strongly agree). High mean indecte high level of knowledge, skills, or attitudes towards patient safety. It consisted of four domains: comfort (5 items), error reporting (8 items), denial (4 items), and culture (5 items) [20]. The inter-item Cronbach’s correlation coefficient of the entire scale was 0.71 [20]. The researchers chose four items to obtain information about patient safety and medical error. The reliability for current study cronbach’s alpha was 0.83.

### Data collection

The researchers contacted faculties’ deans and instructors responsible for teaching students in both universities and explained the purpose of the study and the data collection process. Then, students who expressed interest in participating in the study and met inclusion criteria received a paper cover letter that included information about the study, the purpose, procedure, possible risks, the right to withdraw, confidentiality, anonymity, and consent form. Eligible students were asked to sign informed consent. Then, the authors distributed the self-reported questionnaire to the students between July 7, 2022 and Aug 29, 2022. The duration to complete the questionnaire was 12–15 minutes. The students filled it in quiet rooms in nursing faculties and returned it on the same day to the researchers. The researchers were available during data collection to answer students’ questions and ensure that they understood the items of the questionnaire.

### Ethical consideration

Ethical approvals were obtained from the Institutional Review Board (IRB) of the Faculty of Nursing-Zarqa University (ref.nr: 18/2021). In addition, informed consent was obtained from the students, and the Declaration of Helsinki 1964 ethical principles (World Medical Association 1964) were applied to the study. The researchers informed students that participation in the study is voluntary, and their participation or refusal to participate in the study will not affect their marks in any course during their study. Participants’ confidentiality and anonymity were maintained; the researchers coded the data, and no one else was allowed to access the data other than the researchers.

### Statistical analysis

Data were analyzed using the Statistical Package for the Social Sciences (SPSS) version 26. Descriptive statistics were used to describe participants’ sociodemographic characteristics. The independent samples *t*-test was used to assess the difference in mean knowledge level about QI among nursing undergraduate students from both universities. Spearman’s correlation coefficient was also used to examine the relationship between obtaining information on healthcare quality from undergraduate nursing programs and the level of knowledge about QI. The level of significance was set as 0.05.

## Results

### Sociodemographic characteristics of participants

Overall, 147 students out of 152 students completed the questionnaires. The response rate was (96.7%). Table 1 shows the mean age of the students was 20.7 years (SD = 1.16). Concerning the settings, there were 79 (53.7%) students from the public university and 68 (46.3%) from the private university. The majority of students (46.3%) were in their second year. Regarding the history of being educated about QI, 86 (58.5%) students obtained healthcare quality and QI information from a university program. Also, 87 (59.2%) students responded that they did not obtain information about patient safety from a university program.

**Table 1:** Sociodemographic characteristics (N = 147).

Characteristics	Frequency (%)
**Type of University**	
Public	79 (53.7)
Private	68 (46.3)
**Gender**	
Male	62 (42.2)
Female	85 (57.8)
**Academic Year**	
Second	68 (46.3)
Third	43 (29.3)
Forth	36 (24.5)
**Have you obtained information about quality in healthcare while studying your undergraduate nursing program at the university?**	
Yes	86 (58.5)
No	61 (41.5)
**Have you obtained information about quality improvement while studying your undergraduate nursing program at the university?**	
Yes	86 (58.5)
No	61 (41.5)
**Have you obtained information about patient safety from sources other than the undergraduate nursing program at your university?**	
Yes	60 (40.8)
No	87 (59.2)
**Have you obtained information about quality in healthcare from sources other than the undergraduate nursing program at your university?**	
Yes	43 (29.3)
No	104 (70.7)
**Have you obtained information about quality improvement in healthcare from sources other than the nursing program at your university?**	
Yes	38 (25.9)
No	109 (74.1)

### Knowledge about quality improvement, patient safety, medical error

The students’ overall knowledge of QI score was 57.7 (SD = 22.2). Regarding patient safety, 110 (74.8%) students observed medical errors in the clinical area. Also, 84 (57.1%) of students disclosed a medical error to a faculty member; the same percentage of students did not disclose a medical error to a staff member. However, only 68 (46.3%) students were trained to write incident reports, as presented in Table 2.

**Table 2:** Nursing students’ responses regarding QI and patient safety situation.

Items	Frequency (%)
**Knowledge about QI, M = 57.7; SD = 22.2**	
**Have you observed a medical error in your clinical experiences?**	
Yes	110 (74.8)
No	37 (25.2)
**Have you disclosed a medical error to a faculty member?**	
Yes	84 (57.1)
No	63 (42.9)
**Have you disclosed a medical error to a staff member?**	
Yes	63 (42.9)
No	84 (57.1)
**Have you trained to write an incident report**	
Yes	68 (46.3)
No	79 (53.7)

Note: SD = Standard Deviation, M = Mean, QI = Quality Improvement

### The differences in knowledge of quality improvement based on the type of university

The results of the independent samples *t*-test showed a significant difference in the mean level of knowledge between students from the two universities (*t*(146) = -7.360, *P*≤.001). Students in private university (M = 74.6, SD = 15.2) had highest level of knowledge of quality improvement than students from the public university (M = 44.1, SD = 18.4), as presented in Table 3.

**Table 3:** Differences in the level of knowledge regarding quality improvement.

Type of University	Mean	*SD*	*t*	*P*
**Public**	44.6	17.8	-10.019	≤.001*
**Private**	72.9	16.2		

Note: SD = Standard Deviation, t = t-test statistic, P = Alpha Value

### The relationship between knowledge toward quality improvement and source of obtaining information about quality in healthcare

The Spearman correlation test was used to assess the relationship between the level of knowledge about QI and the source of obtaining information about quality in healthcare, as presented in Table 4. The results showed a significant negative correlation between knowledge level about QI and obtaining information about QI and quality in healthcare from the undergraduate nursing program at the university (*p* ≤.001). This means that students who reported getting more information about QI and quality in healthcare from their undergraduate program actually had lower knowledge levels of QI and quality in healthcare. Also, the analysis revealed a significant positive correlation between knowledge level about QI and obtaining information about QI, quality in healthcare, and patient safety from sources other than the undergraduate nursing program at the university (*p* ≤.001). This indicated that students who gained knowledge about QI, healthcare quality, and patient safety from outside sources tended to have a higher level of understanding about it.

**Table 4:** The relationship between source of obtaining quality in healthcare information and level of knowledge about QI.

Items	Knowledge about Quality Improvement	
	r	p
**Obtained information about quality in healthcare from the undergraduate nursing program at the university**	-.52	≤.001
**Obtained information about quality improvement from the undergraduate nursing program at the university**	-.52	≤.001
**Obtained information about patient safety from sources other than the undergraduate nursing program at the university**	.37	≤.001
**Obtained information about patient safety from sources other than the undergraduate nursing program at the university**	.30	≤.001
**Obtained information about quality improvement from sources other than the undergraduate nursing program at the university**	.50	≤.001

Note: r = Spearman’s correlation Coefficient, P = Alpha Value

## Discussion

The current study aimed to assess undergraduate nursing students’ level of knowledge about QI and patient safety practices and to examine the relationship between the level of knowledge about QI and the source of obtaining information about QI and healthcare quality in Jordan. The results showed that the students’ overall knowledge of QI score was 57.7 (SD = 22.2). This was higher than the knowledge level among nursing students reported in a study conducted in Egypt, in which 36.0% reported having no knowledge about quality improvement [21]. However, the sample consisted of 100 students in their first year at university. This was also higher than reported among nursing students who reported poor QI knowledge and skills; They scored 40.5 out of 80 on QI skills [21]. However, the response rate was only 10% (n = 583).

The present study showed that most students observed medical errors during clinical training. Nevertheless, 57.1% did not disclose medical errors to a staff member. It could be attributed to fear of violating the traditional medical hierarchy; students may have limited knowledge in adequately explaining the nature of medical errors [22, 23]. Moreover, students usually start clinical training at the second year level, and during this year, they may still have limited knowledge about dealing with medical errors properly. It deserves mentioning that only 46.3% of the students in the current study were trained to write incident reports, reflecting insufficient competence among students regarding dealing with incidents and medical errors. This might hinder them from disclosing medical errors. Nevertheless, nursing students usually receive more training about incident reports in the fourth year of their undergraduate study.

The current study’s findings revealed that students from the private university had a higher level of knowledge about QI than public university students. This difference could be attributed to the availability of healthcare-quality courses in the nursing curriculum of the private university. This finding is consistent with the results of previous studies [19, 24–26]. The teaching methods and learning resources differ between the two universities. The financial support for teaching and learning in private universities is more straightforward than in governmental universities. These factors could possibly affect the results and justify this difference in knowledge level about QI.

It was reported that safe patient care could be enhanced by adequate preparation and education of nurses during their undergraduate education [27]. Nurse academicians and educators are responsible for teaching nursing students about safe practice and high-quality care [23]. In addition, accreditation is considered a requirement for healthcare institutions that aim to maintain QI [28]. This makes it crucial for nursing students to gain knowledge about QI during their undergraduate studies to prepare them for future job vacancies.

The results of the current study revealed that students with high levels of knowledge about QI are enrolled in an undergraduate nursing program that does not provide information about QI and quality in healthcare. It was reported that nursing curricula still lack patient safety and quality education, and few research studies investigate patient safety practices among undergraduate students [29]. The current study’s finding contradict what was reported by Maxwell and Wright [19], who used online modules and flipped classrooms to improve nursing students’ knowledge toward quality improvement. However, a few studies assessed the impact of undergraduate nursing courses on students’ knowledge of QI.

The results of the present study indicated that students who obtained healthcare quality information outside the universities had a high level of knowledge about QI information. This consistent with previous studies’ findings [19, 24, 30]. It was revealed previously that undergraduate nursing students’ knowledge regarding QI increased significantly following joining a group work and attending a relevant introductory course [24]. The researchers of the previous study reported that students’ level of knowledge about QI increased significantly from 2.0 (fair) to 3.1 (good) after introducing the intervention. The current study’s findings are congruent with the findings of Pauly-O’Neill and Cooper (2013), who pointed out that engaging nursing students in activities concerning quality improvement, informatics, and evidence-based practice led to increasing their competence in QI.

Quality improvement is vital for health care. Having dynamic and complex healthcare systems necessitates improving patients’ outcomes by providing high-quality and safe care [31]. It was reported that applying quality and safety measures in healthcare can improve patient outcomes and should be integrated into residency programs [32]. The current study’s findings imply that improving nursing students’ knowledge regarding QI and patient safety is a priority. This is expected to enable them to enhance healthcare quality after graduation and start their first clinical job. In addition, nursing academicians are recommended to integrate QI and patient safety concepts in nursing curricula. For example, introducing a mandatory course to undergraduate nursing programs is recommended. Hopefully, this could decrease the level of student stress that could arise from involvement in the accreditation process of healthcare facilities after employment. Besides, conducting short-term courses and activities about QI and patient safety also helps. Furthermore, nursing administrators are recommended to ensure new graduates are engaged in learning about QI once recruited as a part of the general nursing orientation program. Nursing administrators are encouraged to follow up with clinical resource nurses about QI competencies among newly recruited nurses and ensure familiarizing them with patient safety concepts. Moreover, nursing researchers are recommended to conduct a rigorous interventional study to examine the effect of educational programs and undergraduate QI courses on knowledge about QI among undergraduate nursing students. Furthermore, conducting a qualitative research study is recommended to understand the challenges of learning about QI among undergraduate nursing students.

### Study limitations

The current study has some limitations. First, the sample was convenient and was chosen from two universities in the middle of Jordan only. This could negatively affect the study’s external validity and limit the generalizability of the findings. Second, this cross-sectional study does not establish the causal relationship between variables. Third, no information about students’ academic performance were collected, which could affect the level of knowledge. However, the response rate was high, and this study is the first known to assess undergraduate nursing students’ knowledge level about QI in Jordan and one of the fewest studies that addressed this topic globally.

## Conclusions

The present study aimed to assess Jordanian nursing students’ knowledge of QI and examine the relationship between the level of knowledge about QI and obtaining information about QI healthcare quality from different sources. The results showed that nursing students’ overall knowledge about QI scores was moderate. In addition, undergraduate nursing students who studied at a private university and obtained information about QI, healthcare quality, and patient safety from sources other than the undergraduate nursing program had a higher level of knowledge about QI than nursing students who studied at a public university and did not obtain such information. Teaching students about QI and patient safety is crucial to enable nurses to provide safe and competent care. For this reason, having undergraduate courses and education initiatives about QI and patient safety is the initial step for improving the quality and safety of patient healthcare.

## References

[1] Kruk ME, Gage AD, Arsenault C, Jordan K, Leslie HH, Roder-DeWan S, et al. High-quality health systems in the Sustainable Development Goals era: time for a revolution. The Lancet Global Health. 2018; 6(11): e1196–e252. doi: 10.1016/S2214-109X(18)30386-3 30196093 PMC7734391

[2] Ta’an WaF, Suliman MM, Al‐Hammouri MM, Ta’an A. Prevalence of medical errors and barriers to report among nurses and nursing students in Jordan: A cross‐sectional study. Nursing Forum. 2021; 56(2): 284–90. doi: 10.1111/nuf.12542 33336425

[3] Khamaiseh A, Al-Twalbeh D, Al-Ajlouni K. Patient safety culture in Jordanian primary health-care centres as perceived by nurses: a cross-sectional study. East Mediterr Health J. 2020; 26(10): 1242–50. doi: 10.26719/emhj.20.044 33103752

[4] Lee SE, Morse BL, Kim NW. Patient safety educational interventions: A systematic review with recommendations for nurse educators. Nursing Open. 2022; 9(4): 1967–79. doi: 10.1002/nop2.955 34047058 PMC9190690

[5] Al-Oweidat IA, Saleh A, Khalifeh AH, Tabar NA, Al Said MR, Khalil MM, et al. Nurses’ perceptions of the influence of leadership behaviours and organisational culture on patient safety incident reporting practices. Nurs Manag (Harrow). 2023; 30(6): 33–41. doi: 10.7748/nm.2023.e2088 37190777

[6] World Health Organization. Global patient safety action plan 2021–2030: towards eliminating avoidable harm in health care. Geneva: World Health Organization 2021:86.

[7] World Health Organization. Global patient safety action plan 2021–2030: towards eliminating avoidable harm in health care: World Health Organization, 2021.

[8] World Health Organization. Patient safety incident reporting and learning systems: technical report and guidance. Geneva: World Health Organization 2020.

[9] Hopkins JJB. Medical Error: The Third Leading Cause of Death in the US. 2016; 353. 10.1136/bmj.i2139 27143499

[10] Rodziewicz T, Houseman B, Hipskind J. Medical Error Reduction and Prevention. StatPearls. Treasure Island (FL). StatPearls Publishing Copyright 2021. 29763131

[11] Lee Adler DY, Li M, McBroom B, Hauck L, Sammer C, Jones C, et al. Impact of inpatient harms on hospital finances and patient clinical outcomes. 2018; 14(2): 67. doi: 10.1097/PTS.0000000000000171 25803176 PMC5965919

[12] Halperin O, Bronshtein O. The attitudes of nursing students and clinical instructors towards reporting irregular incidents in the medical clinic. Nurse Education in Practice. 2019; 36: 34–9. doi: 10.1016/j.nepr.2019.02.018 30851637

[13] Vaismoradi M, Tella S, P AL, Khakurel J, Vizcaya-Moreno F. Nurses’ Adherence to Patient Safety Principles: A Systematic Review. Int J Environ Res Public Health. 2020; 17(6). doi: 10.3390/ijerph17062028 32204403 PMC7142993

[14] Dimitriadou M, Merkouris A, Charalambous A, Lemonidou C, Papastavrou E. The knowledge about patient safety among undergraduate nurse students in Cyprus and Greece: a comparative study. BMC Nurs. 2021; 20(1): 110. doi: 10.1186/s12912-021-00610-6 34172054 PMC8234646

[15] Bressan V, Causero G, Stevanin S, Cadorin L, Zanini A, Bulfone G, et al. Nursing Students’ Knowledge of Patient Safety and Development of Competences Over their Academic Years: Findings from a Longitudinal Study. Slovenian Journal of Public Health. 2021; 60: 114–23. doi: 10.2478/sjph-2021-0017 33822834 PMC8015659

[16] Suliman M JNep. Measuring patient safety competence among nursing students in the classroom and clinical settings. 2019; 40(3): E3–E7. doi: 10.1097/01.NEP.0000000000000460 30920475

[17] Buchner A, Erdfelder E, Faul F, Lang A. G* Power 3.1 manual. 3.1.9.7 ed. Düsseldorf, Germany: Heinrich-Heine-Universitat Dusseldorf 2020.

[18] Dycus P, McKeon L. Using QSEN to measure quality and safety knowledge, skills, and attitudes of experienced pediatric oncology nurses: an international study. Quality Management in Healthcare. 2009; 18(3): 202–8. doi: 10.1097/QMH.0b013e3181aea256 19609190

[19] Maxwell KL, Wright VH. Evaluating the Effectiveness of Two Teaching Strategies to Improve Nursing Students’ Knowledge, Skills, and Attitudes About Quality Improvement and Patient Safety. Nursing education perspectives. 2016; 37(5): 291–2. doi: 10.1097/01.NEP.0000000000000043 27740566

[20] Chenot TM, Daniel LG. Frameworks for patient safety in the nursing curriculum. Journal of Nursing Education. 2010; 49(10): 559–68. doi: 10.3928/01484834-20100730-02 20669876

[21] Ahmed H, Aziz EA, Fathy Y. Perception of patient safety, quality improvement and nursing errors issues among undergraduate nursing students in Faculty of Nursing, Alexandria University, Egypt. IOSR Journal of Nursing and Health Science (IOSR-JNHS). 2017; 6(3): 83–95.

[22] Rajendran PR. Ethical issues involved in disclosing medical errors. Jama. 2001; 286(9): 1078-. 11559295 10.1001/jama.286.9.1078

[23] Cooper EE. Nursing students’ perception of safety in clinical settings: From the quality and safety officer. Journal of Nursing Education and Practice. 2017; 7(10): 91–7. doi: 10.5430/jnep.v7n10p91

[24] Kyrkjebø J, Hanssen T, Haugland BØ. Introducing quality improvement to pre-qualification nursing students: evaluation of an experiential programme. BMJ Quality & Safety. 2001; 10(4): 204–10. doi: 10.1136/qhc.0100204.. 11743148 PMC1743458

[25] Dotson BJ, Lewis L. Teaching the quality improvement process to nursing students. Journal of Nursing Education. 2013; 52(7): 398–400. doi: 10.3928/01484834-20130613-01 23758156

[26] White KM, Walrath JM. An innovative approach to quality and safety education for baccalaureate nursing students. Annual Review of Nursing Education. 2008; 6: 65.

[27] ANA. The essentials of baccalaureate education for professional nursing practice. 2015.

[28] AlKhenizan A, Shaw C. Assessment of the accreditation standards of the Central Board for Accreditation of Healthcare Institutions in Saudi Arabia against the principles of the International Society for Quality in Health Care (ISQua). Annals of Saudi medicine. 2010; 30(5): 386. doi: 10.4103/0256-4947.67082 20697166 PMC2941252

[29] Mansour M. Current assessment of patient safety education. British Journal of Nursing. 2012; 21(9): 536–43. doi: 10.12968/bjon.2012.21.9.536 22585267

[30] Pauly-O’Neill S, Cooper EE. Addressing gaps in quality and safety education during pre-licensure clinical rotations. 2013.

[31] DeBourgh GA. Synergy for patient safety and quality: academic and service partnerships to promote effective nurse education and clinical practice. Journal of Professional Nursing. 2012; 28(1): 48–61. doi: 10.1016/j.profnurs.2011.06.003 22261605

[32] Morrison RJ, Bowe SN, Brenner MJ. Teaching quality improvement and patient safety in residency education: strategies for meaningful resident quality and safety initiatives. JAMA Otolaryngology—Head & Neck Surgery. 2017; 143(11): 1069–70. doi: 10.1001/jamaoto.2017.1317 28796866

